# Silica@zirconia Core@shell Nanoparticles for Nucleic Acid Building Block Sorption

**DOI:** 10.3390/nano11092166

**Published:** 2021-08-25

**Authors:** Livia Naszályi Nagy, Evert Dhaene, Matthias Van Zele, Judith Mihály, Szilvia Klébert, Zoltán Varga, Katalin E. Kövér, Klaartje De Buysser, Isabel Van Driessche, José C. Martins, Krisztina Fehér

**Affiliations:** 1 NMR and Structure Analysis Research Group, Department of Organic and Macromolecular Chemistry, Ghent University, Krijgslaan 281 S4, B-9000 Ghent, Belgium; lnaszalyi@gmail.com (L.N.N.); jose.martins@ugent.be (J.C.M.); 2 Sol-Gel Centre for Research on Inorganic Powders and Thin Films Synthesis, Department of Chemistry, Ghent University, Krijgslaan 281 S3, B-9000 Ghent, Belgium; Evert.Dhaene@UGent.be (E.D.); Matthias.VanZele@ugent.be (M.V.Z.); Klaartje.DeBuysser@ugent.be (K.D.B.); Isabel.VanDriessche@ugent.be (I.V.D.); 3 Institute of Materials and Environmental Chemistry, Research Centre for Natural Sciences, Eötvös Loránd Research Network (IMEC RCNS ELKH), Magyar Tudósok Körútja 2, H-1117 Budapest, Hungary; mihaly.judith@ttk.mta.hu (J.M.); klebert.szilvia@ttk.hu (S.K.); varga.zoltan@ttk.mta.hu (Z.V.); 4 Department of Inorganic and Analytical Chemistry, University of Debrecen, Egyetem tér 1, H-4032 Debrecen, Hungary; kover@science.unideb.hu; 5 Molecular Recognition and Interaction Research Group, Hungarian Academy of Sciences-Eötvös Loránd Research Network at University of Debrecen, Egyetem tér 1, H-4032 Debrecen, Hungary

**Keywords:** nanocarrier, nanoparticle, age-dependent adsorption, Langmuir isotherm, deoxynucleoside monophosphate, silica@zirconia, core@shell, solution NMR, buffer interference with adsorption

## Abstract

The development of delivery systems for the immobilization of nucleic acid cargo molecules is of prime importance due to the need for safe administration of DNA or RNA type of antigens and adjuvants in vaccines. Nanoparticles (NP) in the size range of 20–200 nm have attractive properties as vaccine carriers because they achieve passive targeting of immune cells and can enhance the immune response of a weakly immunogenic antigen via their size. We prepared high capacity 50 nm diameter silica@zirconia NPs with monoclinic/cubic zirconia shell by a green, cheap and up-scalable sol–gel method. We studied the behavior of the particles upon water dialysis and found that the ageing of the zirconia shell is a major determinant of the colloidal stability after transfer into the water due to physisorption of the zirconia starting material on the surface. We determined the optimum conditions for adsorption of DNA building blocks, deoxynucleoside monophosphates (dNMP), the colloidal stability of the resulting NPs and its time dependence. The ligand adsorption was favored by acidic pH, while colloidal stability required neutral-alkaline pH; thus, the optimal pH for the preparation of nucleic acid-modified particles is between 7.0–7.5. The developed silica@zirconia NPs bind as high as 207 mg dNMPs on 1 g of nanocarrier at neutral-physiological pH while maintaining good colloidal stability. We studied the influence of biological buffers and found that while phosphate buffers decrease the loading dramatically, other commonly used buffers, such as HEPES, are compatible with the nanoplatform. We propose the prepared silica@zirconia NPs as promising carriers for nucleic acid-type drug cargos.

## 1. Introduction

With the rapid emergence of third-generation vaccines, nucleic acids are increasingly deployed both as coding for antigenic proteins as well as adjuvants in immunity-inducing preparations. This strategy has been particularly successful in fighting the worldwide epidemic caused by SARS-CoV-2, for which several nucleic acid-based vaccines have been developed in a spectacularly short amount of time, with multiple further preparations in the pipeline [1]. In third-generation vaccines, antigens are encoded by genetically engineered plasmid DNA in DNA vaccines [2] or by messenger RNA in mRNA vaccines [3], both of which induce expression of the target protein in the cell in situ. Nucleic acid type adjuvants [4,5,6], such as CpG motif-containing oligodeoxynucleotides (CpG ODNs) [7], are sequences with immune-modulating properties and are needed to induce and direct the immune response of the vaccine. However, nucleic acid delivery is not only relevant in prophylactic vaccines against viral diseases but also in the development of therapeutic cancer vaccines [8] and in gene therapies [9].

Due to their polyanionic nature, nucleic acids cannot easily permeate the cell membrane, and they are susceptible to enzymatic degradation by nucleases. For these reasons, therapeutic nucleic acid molecules can vastly benefit from delivery systems. Indeed, one of the major stumbling blocks for the mass deployment of SARS-CoV-2 mRNA vaccines is the requirement of ultra-low temperature storage needed for their stability in cationic lipid nanoparticle formations [1]. Since DNA and RNA are negatively charged, delivery systems based on electrostatic interactions for immobilization can be used for both types of molecules. Particle-mediated in vivo delivery options [10] includes cationic lipids and polymers, cell-penetrating peptides and biological and inorganic particles. [11] The development of suitable nanocarriers for the immobilization of nucleic acids requires cheap, non-toxic, biodegradable materials available in large quantities that are able to adsorb nucleoside–phosphate-containing molecules with high capacity. Furthermore, the process leading to the nanocarriers should be controllable, reproducible and up-scalable to be of industrial interest. The size of the nanocarrier is of importance as the 20–200 nm size range was shown to enhance the immunogenic effect of the vaccine formulation [12].

Zirconia is a material studied extensively as a dental and orthopedic implant coating because of its biocompatibility and favorable osseointegration [13]. Some research groups studied its potential as a drug delivery nanocarrier [14,15,16]. However, as the size and shape control of sol–gel-derived pure and colloidally stable zirconia particles has only been achieved for diameters <10 nm [17,18,19,20] and >200 nm [21], researchers started to use organic (such as bacterial or polymeric) or inorganic templates for zirconia deposition [15,22,23,24,25,26,27]. In the last step of the template removal, these procedures all apply a thermal treatment to the particles, which was, however, shown to lower the amount of ligand-adsorbing sites on the surface of zirconia due to recrystallization into a tetragonal form and decrease the number of surface defects [16,28]. Moreover, as we were aiming at the preparation of core@shell particles in the 20–100 nm diameter range, which is smaller than those formerly described in the literature, it was crucial to avoid the sintering effect. Thus, here in our wet chemical synthetic route, very mild conditions were used to avoid recrystallization and sintering, and extreme care was taken to control the colloidal stability of the resulting materials.

**Scheme 1:**
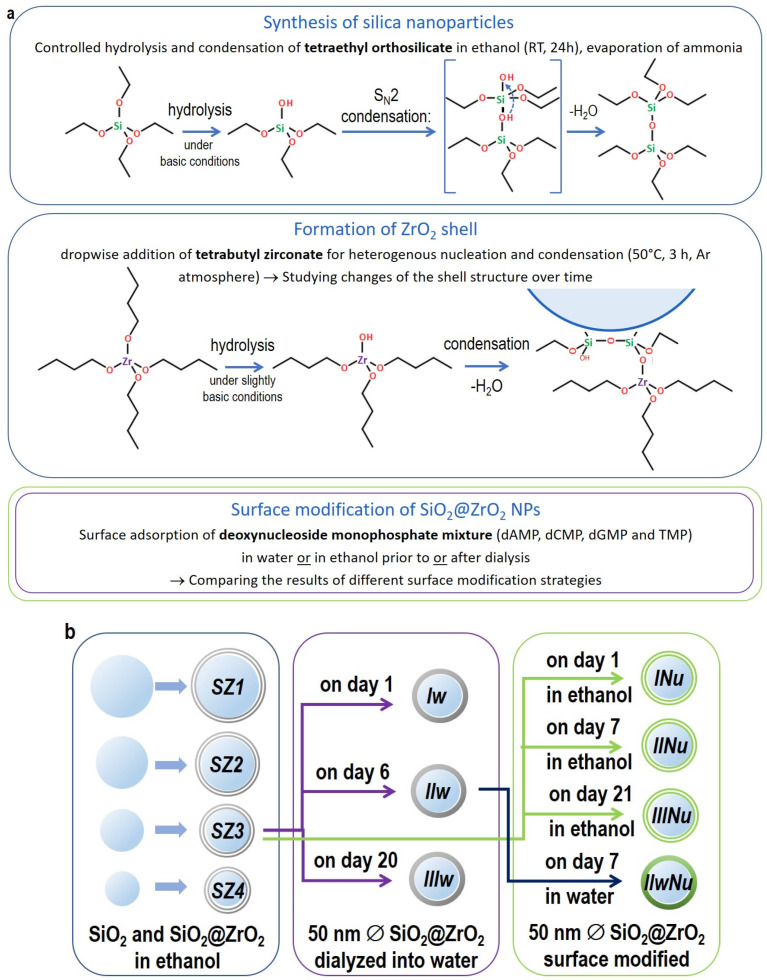
A summary of the chemical synthesis steps (**a**) and sorption optimization strategy (**b**). According to our strategy, the optimal nanocarrier size was chosen first to achieve the highest possible specific surface area (**left panel**), then the optimal aging time before transfer into the water was defined to get maximal stability of the shell (**middle panel**); finally, the best surface modification strategy, leading to the highest cargo was chosen by the characterization of the four surface-modified samples (**right panel**). The silica core is depicted in blue; the deposited zirconia shell is in grey and the adsorbed dNMPs are shown in green. Batches ***SZ1***, ***SZ2***, ***SZ3*** and ***SZ4*** are native silica@zirconia NPs with increasing sizes in the 10-100 nm range; samples ***Iw***, ***IIw*** and ***IIIw*** are NPs transferred into water by dialysis at day 1, 7 and 21, respectively; samples ***INu***, ***IINu*** and ***IIINu*** are NPs modified using the ethanolic surface modification procedure at the age of 0, 6 and 20 days after dialysis, and finally, the ***IIwNu*** sample is treated by the aqueous surface modification procedure 7 days after synthesis.

As a proof of concept, we aimed at the elucidation of the optimal conditions for DNA building blocks using deoxynucleoside monophosphate (dNMP) adsorption at the surface of the silica@zirconia core@shell NPs. The steps of the chemical synthesis are visualized in Scheme 1a. The strategy used for the optimization of dNMP sorption is shown in Scheme 1b.

First, we optimized the synthetic conditions of the core@shell nanoparticles for the different size ranges (left panel in Scheme 1b) and characterized their morphology and size by transmission electronmicroscopy (TEM) and dynamic light scattering (DLS). We studied the time-dependent structural changes of the native NPs during the condensation step of the zirconia deposition. TEM and nuclear magnetic resonance (NMR) revealed details of the evolution of the zirconia shell composition showing a physisorbed layer of zirconia starting material on the surface in addition to a solid zirconia shell. X-ray diffraction (XRD) also indicated changes over time in the composition of the crystalline phases. Fourier-transform infrared (FTIR) gave insight into the number and the state of the potential surface binding sites via monitoring the surface attached carbonate species [16,29] by quantifying the number of hydrogenocarbonate, monodentate and bridged carbonate molecules in the solution of the synthesis.

In the following, we monitored the behavior of the particles during and after dialysis into the water (the middle panel in Scheme 1b). DLS measurements and TEM pictures were used to monitor aggregation upon dialysis into the water for samples with different aging times of the zirconia shell. FTIR and NMR spectroscopies were indispensable for characterizing the surface chemistry at the solid-solution interface. We used the NMR toolbox [30] to confirm the presence of a physisorbed layer of zirconia starting material on the surface, which rapidly hydrolyzed upon dialysis into water and was found to be a major determinant of colloidal stability versus aggregation during this process. Zeta potential versus pH curves of the native NPs established the pH ranges, in which the particles are electrostatically stabilized by surface charges. FTIR revealed that the smallest size NPs did not retain their zirconia shell by monitoring the silica vibrational bands and also provided further insight into the overall adsorption capacity of the surface and the acid-base nature of the adsorption sites after transfer into the water.

We intended to understand how and under which conditions the adsorption takes place (right panel of Scheme 1b) and how it affects the colloidal stability of the particles. A shift of the isoelectric point (IEP) of the NPs under the effect of dNMP chemisorption in combination with the determination of the free ligand content in the solution revealed that the adsorption of ligands is favored at acidic pH. This enabled us to pinpoint the pH range optimal for both colloidal stability and ligand binding. We compared different surface modification methods by DLS, TEM, FTIR and zeta potential measurements and also studied the effect of the aging time of the zirconia shell onto the adsorption.

In view of the desired biological application, we inspected the effect of several buffers, such as phosphate, HEPES, PIPES, MES, MOPS and MOPSO—usually referred to as non-interfering buffers [31]—on the adsorption phenomenon. We established the adsorption isotherms of dNMP equimolar mixtures at the surface of the silica@zirconia NPs in four of the chosen buffers and derived the maximum adsorption capacity at pH 7.4 under equilibrium conditions. In parallel, we measured the dNMP content of 50 nm diameter nanocarriers surface modified using two different procedures by thermogravimetric analysis (TGA). In this way, we could define the optimal procedure for the surface modification of the nanocarriers whereby the dNMP load was found to be very close to the theoretical maximum obtained by Langmuir isotherms. The zirconia surface showed microporosity as determined by N_2_ adsorption-desorption isotherms, which were also used to determine specific surface areas.

As a result of this development process, we were able to establish the optimum conditions for a procedure that yielded colloidally stable silica@zirconia NPs with the ability to absorb DNA-type molecules with high capacity.

## 2. Experimental

### 2.1. Materials

Tetraethyl orthosilicate (TEOS, 99%, abcr), ammonia solution (fresh, 32%, EMPLURA, Merck, Darmstadt, Germany), absolute ethanol (LiChrosolv^®^, gradient grade for liquid chromatography, water content <0.1%, Merck, Darmstadt, Germany), zirconium(IV) butoxide solution (TBOZ, 80% in n-butanol, Sigma-Aldrich, St. Louis, MO, USA, kept under argon,) were used for the NP synthesis. The biomolecules adsorbed at the surface of the NPs were: 2′-deoxyadenosine-5′ monophosphate (dAMP, 98–100%, Sigma grade, 2′-deoxycytidine-5′ monophosphate (dCMP, ≥95.0%, Sigma grade), 2′-deoxyguanosine-5′ monophosphate disodium salt hydrate (dGMP, ≥98.0%, Aldrich), thymidine-5′ monophosphate disodium salt hydrate (TMP, ≥99.0%, Sigma) and 2′-deoxyadenosine monohydrate (≥99%, Sigma-Aldrich, St. Louis, MO, USA).

High-purity deionized water (Sartorius, Göttingen, Germany, Arium 611, 18.2 MΩ·cm) basified with 1 M KOH solution (Titripur^®^ Reag. Ph Eur, Reag. USP, EMD Millipore, Burlington, MA, USA) was used for the transfer of NPs into the water by dialysis. Buffers were prepared using 3-(N-morpholino) propane sulfonic acid (MOPS, BioPerformance certified, >99.5%, Sigma-Aldrich, St. Louis, MO, USA), β-hydroxy-4-morpholinepropanesulfonic acid (MOPSO, Pharma grade, Sigma-Aldrich, St. Louis, MO, USA), 1,4-piperazinediethanesulfonic acid (PIPES, Bioperformence certified, Sigma-Aldrich, St. Louis, MO, USA), 2-(N-morpholino) ethane sulfonic acid hydrate (MES, 99.5%, Sigma-Aldrich, St. Louis, MO, USA), 4-(2-hydroxyethyl)piperazine-1-ethanesulfonic acid (HEPES, 99.5%, Sigma-Aldrich, St. Louis, MO, USA), potassium phosphate dibasic trihydrate (99+%, Acros Organics, Waltham, MA, USA) and potassium phosphate monobasic (p.a., Acros Organics, Waltham, MA, USA). Deuterium oxide was added to samples prior to NMR measurements (99.96%, Euriso-top, Tewksbury, MA, USA).

### 2.2. Preparation of Silica@zirconia NPs

Silica sols of different particle sizes were prepared by the Stöber method [32]. Briefly, 125 mL abs. ethanol was poured in a tall form beaker, and 7.0, 6.0, 5.0 or 3.5 mL ammonia solution was added. The mixture was stirred at 330 rpm covered with parafilm. 5.0 mL TEOS was quickly added, and the reaction mixture was being stirred at room temperature for 24 h. Thereafter, the parafilm was discarded, and additional ethanol (~70 mL) added to the sample prior to the removal of ammonia at 70 °C under vigorous stirring in a ventilated fume hood. The pH of the ethanolic mixture was controlled with a wet pH paper, and the evaporation of ammonia was stopped at pH 8. The solid content of the sample was determined on 3 × 1 mL sol dried out at 85 °C, 10 h. The sol was stored at 4 °C.

The deposition of zirconia at the surface of 100 nm diameter silica cores was carried out by a controlled hydrolysis-condensation of the metal organic precursor. The process was based on Kim and coworkers’ method [33,34]. We applied 8-fold dilution to 50 mL of the silica sol and put the ethanolic reaction mixture under argon bubbling and stirring at 50 °C. Based on TEM mean diameters, 0.11 mmol TBOZ/m^2^ silica surface was added to 25 mL ethanol in the dropping funnel, and dropwise addition was performed. After 2.0 h, the reaction mixture was cooled down and stored at 4 °C. In our optimized procedure for 50 nm diameter NPs, 10-fold dilution was found to be more suitable, and 4–7 days of aging was applied prior to the surface modification or the dialysis into water.

### 2.3. Solvent Exchange and Surface Modification of the Silica@zirconia NPs

In the case of dAMP, 2 mg/mL ligand solutions were prepared in water using heating to reach complete dissolution of the solid material. The four-component mixture of dNMPs was obtained by adding equal volumes of dAMP, dCMP, dGMP and TMP. The native pH of dNMP solutions were: dAMP (3.1), dCMP (3.4), dGMP (8.2), TMP (7.8) and dNMP mixture (4.9). For controlled adsorption conditions, the pH of all the ligand solutions and mixtures were set to 6.0 ± 0.1.

#### 2.3.1. Surface Modification in Ethanol

The ligand was added to an aliquot of the freshly prepared silica@zirconia core@shell sol at a ligand-to-NP surface ratio of 0.8 µmol/m^2^ to 22.7 µmol/m^2^ (calculation based on the TEM mean particle size). The excess of ligands was removed by dialysis against pure or neutralized water (CelluSep dialysis tubing, MWCO 10.000 Da, 50 mm flat width).

#### 2.3.2. Surface Modification in Water

An aliquot of the ethanolic core@shell sol was filled into a dialysis bag and was dialyzed against cold (4–8 °C), basified pure water (pH 8.5–9.5). The ligand solution was added in a ratio of 50 µmol/m^2^, and further dialysis was done against pure water.

### 2.4. Characterization

Morphological investigations of the NPs were carried out on a JEOL JEM-2200FS transmission electron microscope operated at 200 kV with a Cs corrector. Diluted samples were dropped and dried on holey carbon-coated copper grids (200 mesh).

DLS and zeta potential measurements were performed at 25 °C on a Malvern Zetasizer Nano ZS (Malvern, Worcs, UK) equipped with He-Ne laser (λ = 633 nm) and backscatter detector at a fixed angle of 173°. The pH of the samples was measured using an extended length pH electrode with micro-bulb together with a 9126 pH/ORP meter (HANNA Instruments Ltd., Leighton Buzzard, UK). The parameters used for the measurement were: RI_SiO2_ = 1.475, RI_SiO2@ZrO2_ = 2.152, absorption for both oxides: 0.001, RI_ethanol_ = 1.364, RI_water_ = 1.330, Viscosity_ethanol_ = 1.0740 mPa.s, Viscosity_water_ = 0.8872 mPa.s and dielectric constant_water_ = 78.5.

The solid content of the samples was assessed on 3 × 1 mL aliquots dried out at 85 °C (for samples in ethanol) or 95 °C for samples in water in a Binder ED53 drying oven.

FTIR absorption spectrum was recorded using a Perkin-Elmer Frontier MIR Spectrometer equipped with a deuterated triglicine sulfate (DTGS) detector and a single reflection attenuated total reflection (UATR) unit (SPECAC “Golden Gate”) with diamond ATR element. Records of 32 scans and a 4 cm^−1^ resolution were applied.

One-dimensional and two-dimensional NMR spectra were recorded on a Bruker Avance II 700 MHz (frequency for protons) NMR spectrometer equipped with a Prodigy cryoprobe. During sample preparation, we applied samples with an 8-fold ligand to NP ratio using an Eppendorf centrifuge 5415R (14.000 rpm, 5 min), followed by the addition of 10% D_2_O and filling into 5 mm diameter quartz tubes or Shigemi tubes.

Powder X-ray diffraction patters of the adsorbents were collected on a Thermo Scientific ARL XTra diffractometer, operated at 40 kV, 40 mA using Cu K_α_ radiation of 0.15418 nm wavelength. Pas Academic V4.1 software was used for Rietveld refinement.

The Brunauer–Emmett–Teller (BET) specific surface area and micropore volume were evaluated from the N_2_ adsorption-desorption isotherm. Nitrogen physisorption measurement was performed at 77 K (−196 °C) using a static volumetric apparatus (Quantachrome Autosorb 1C analyzer). The samples were previously degassed at 373 K (100 °C) for 24 h. Nitrogen adsorption data were obtained using ca. 0.05 g of sample and successive doses of nitrogen until p/p_0_ = 1 relative pressure was reached. Only the nitrogen adsorption volumes up to a relative pressure of 0.1 were considered in the micropore size distribution. The micropore volume was obtained from the DR (Dubinin–Radushkevich) plot.

TGA was performed in a NETZSCH STA 449 F3 Jupiter instrument. Powder samples were heated to 800 °C at a rate of 10°/min in air flow.

The adsorption of biomolecules to the silica@zirconia NP surface was monitored by zeta potential measurements of the particulate samples complemented by the UV-visible study of their supernatant as a function of the pH. First, a large batch of native NP suspension was prepared in water with a solid content set to 1 mg/mL. For each run, 10 mL aliquot of this suspension was added 2 or 3 µmol/m^2^ ligand solution (and eventually 10 mM buffer) in a stirred vessel. The pH of the suspension was controlled in the range of pH 9–4 using 1 M NaOH and 1 M HCl, and at every step, a 730 µL sample was placed in a disposable folded capillary cell. After recording the zeta potential in 3 measurements, the sample was transferred into an Eppendorf tube for centrifugation at 12.000 rpm, 3 min. Finally, the UV spectrum of the supernatant was recorded in 5-fold dilution in quartz cuvette in the wavelength range of 200–400 nm (Perkin-Elmer, Waltham, MA, USA, Lambda 950 spectrometer, Deuterium lamp, slit width: 2 nm, resolution: 2 nm).

The adsorption isotherms were determined on the supernatants of 1 mL samples containing 0.45 mg/mL NP, 20 mM buffer and 4, 12, 16, 20, 24, 28, 32, 40, 80, 120, 160 and 200 µg/mL dNMP mixture. The equilibration was done at room temperature for 24 h. The NPs were centrifuged at 12.400 rpm for 10 min, and the supernatants were collected. Finally, 2-, 5- and 10-fold dilutions were applied according to the concentration of the dNMP in the solution. The same dilutions were used for the buffer as background.

## 3. Results

### 3.1. Native Silica@zirconia Core@shell NPs

In order to obtain high adsorption capacity nanocarriers, we gradually increased the specific surface area of our samples by reducing the size of the Stöber silica cores from 81 ± 12 nm to 13 ± 2 nm resulting in batches ***SZ1*** to ***SZ4*** (Table 1). We adapted the synthetic procedure of the zirconia deposition accordingly. Keeping the original n_TBOZ_/SiO_2_ surface ratio was optimal for smaller particle sizes of batches ***SZ1*** and ***SZ2***, and on the other hand, the optimal dilutions for ***SZ3*** and ***SZ4*** were found to be 10 and 12, respectively.

We summarized the mean size and size distribution properties of the core and core@shell NPs as evaluated by DLS and TEM investigations in Table 1. The NPs were spherical, and the samples showed a relatively narrow size distribution. TEM pictures and DLS size distribution functions are shown in Figures S1 and S2 in Supplementary Materials.

According to our experience, in the second reaction step, it was crucial to stop the deposition of TBOZ after 2.0 h and cool it to 4 °C. The formation of the zirconia shell was, however, not complete at this time point. As Arnal et al. also observed [22], the “aging” of the reaction mixture was important: they aged their particles for 3 days prior to the removal of the silica core but provided no explanation for this treatment. We decided to study the structural changes occurring in this time period using TEM, FTIR and XRD measurements.

**Figure 1:**
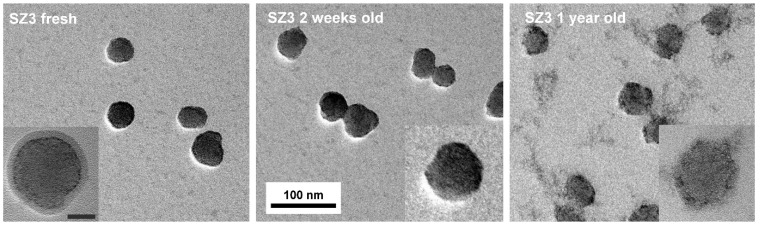
TEM pictures of batch ***SZ3*** in ethanol showing an incomplete condensation of the zirconia precursor on the day after addition to the silica cores (**left**), completed ZrO_2_ shell formation and beginning of the ripening at the age of 2 weeks (**middle**) and the advanced ripening of the 1-year-old core@shell NPs leading to the dissolution-redeposition of the material next to the NPs (**right**).

TEM pictures of the one-day-old core@shell NPs revealed that in addition to a very thin shell of crystalline ZrO_2_ (high contrast contour) surrounding the silica sphere, there is a thicker amorphous or polymer-like deposit on the surface of the NPs (Figure 1, left). The surface of the deposit is smooth, and its contrast is much lower than expected for crystalline zirconia. We suggest that this is a physisorbed multilayer of TBOZ that condensates slowly to give ZrO_2_. For a two-week-old sol, only the high-contrast thin shell can be seen on the surface of the NPs (Figure 1, middle). At this time point, the ripening of the sample has begun leading to dissolution-redeposition of the oxides next to the silica@zirconia core@shell NPs (see the advanced stage for a seemingly stable 1-year-old sample in Figure 1, right).

The broad bands in the XRD spectra (see the example XRD spectrum of a 2-week-old ***SZ3*** sample shown in Figure S3 in Supplementary Materials) of differently aged ***SZ3*** batches evidenced a small crystallite size of ca. 5 nm. The Rietveld analysis showed that freshly prepared ***SZ3*** presented purely monoclinic zirconia, while 36% of zirconia adopted a cubic crystalline phase one week later (Table 2). This does not appear to be in full agreement with the phase diagram of pure zirconia, according to which the cubic crystalline phase is not stable at room temperature [35]. While metallic dopants (Ca^2+^, Y^3+^, etc.) were found to lower the temperature for the formation of cubic ZrO_2_, high annealing temperatures are, in general, required for its formation (800–1000 °C). “Low-temperature” syntheses of pure, cubic ZrO_2_ were described by Prakashbabu [36] and Salavati-Niazari [37], using organic ligands to fix the precursor’s symmetry during the thermal degradation process at 400 °C and 245 °C. Our hypothesis is that due to the high purity of the system, the Stöber silica surface serves as a scaffold that induces and stabilizes the formation of cubic zirconia. This was supported by the observation of decreasing amount of cubic zirconia during the ripening: when zirconia left the surface, it redeposited under monoclinic crystalline phase (or amorphous form) next to the NPs.

Although FTIR spectroscopy cannot give direct information on ZrO_2_ as its vibrational bands appear at 500 cm^−1^, it allows monitoring surface attached carbonate species that occupied potential ligand-binding sites. It has been previously shown [16,29] that soon after deposition, the most basic sites on the surface of zirconia are immediately binding CO and CO_2_ from the air, thus covering accessible adsorption sites in the form of hydrogenocarbonate, alkaline monodentate carbonate and bridged carbonate. We monitored the ratio of hydrogenocarbonate and carbonate species adsorbed at the particles’ surface, which changed slightly over the observed time. In order to view these changes, we normalized the raw spectra to the Si-O-Si vibrational band at 1090 cm^−1^ (Figure S4 in Supplementary Materials). The intensity of the hydrogenocarbonate (HCO_3_^−^) vibrational band at 1621 cm^−1^ decreased after the first day, while monodentate carbonate (m-CO_3_^2−^) vibrations (1568 cm^−1^) showed a slight increase in intensity starting with day 2. The vibrational bands of bridged carbonate (br-CO_3_^2−^) became more prominent on day 3, with a shoulder appearing at 1549 cm^−1^ and 1393 cm^−1^. These changes evidenced an evolution of the zirconia surface structure during the days following the deposition reaction. The observed carbonate species appear to reflect the acidity of the zirconia surface: at first, acidic hydrogenocarbonate is prominent, which is then converted into more alkaline monodentate and bridged carbonate species as the zirconia layer is ripening.

As a summary of the above TEM, XRD and FTIR analyses, we propose that the transformation of physisorbed TBOZ to zirconia takes several days (4–7), which is deposited first as a mixture of amorphous and monoclinic crystalline states, and then the silica surface induces the formation of cubic zirconia. Furthermore, at this stage, the adsorption sites of the deposited zirconia are occupied by various carbonate species, which are evolving from acidic to more alkaline sites over time.

### 3.2. Transfer of Native Silica@zirconia Core@shell NPs into Water by Dialysis

In view of the biological application, we transferred the NPs of different sizes into the water to study their structural changes and colloidal stability. We have chosen dialysis as a gentle solvent exchange technique to avoid the adverse effects of centrifugation on particle size distribution. Even with dialysis, we observed an increase in DLS particle sizes after solvent exchange, indicating aggregation, particularly when the age of the sol, the temperature and the pH of the dialysis water were not controlled. Regarding the temperature of the dialyzing water, using cold water was found to be crucial for the maintenance of the original NP structure: we used 4–8 °C water changed every 2 h. As to the pH of the dialyzing water and the age of the sol, we carried out dialysis of the ethanolic sol after different aging times, and as expected, both parameters had a significant effect on the properties of the resulting material as follows.

Freshly prepared native NPs (***Iw***) underwent immediate, irreversible aggregation when dialysis against water started, independently of the pH of the water. This was evidenced by the whitening and sedimentation of the sample. Since the agglomerates were no longer in suspension, evaluation of the size distribution of these samples by DLS was not possible. Our interpretation based on the above hypothesis of the zirconia shell formation was that upon addition of water to ***Iw*** during dialysis, a large amount of the remaining physisorbed TBOZ suddenly converts to zirconia, which crystallizes on the surface and connects colliding particles by generating chemical bonds between them.

Using 4–7-day-old native NPs, the sol was still whitened during dialysis (***IIw***), but this process was slow, and—what is more interesting—depending on the pH, a spontaneous redispersion of the NPs occurred in 1–2-weeks’ time at neutral-basic pH. Setting the pH of the dialyzing water to 8.5–9.5 reduced significantly the aggregation resulting in slight or no whitening of the sample. DLS showed predominantly primary particles after 4 days of aging time, while 7-day old particles increased in size with a broader distribution (Figure S5 in Supplementary Materials). Our interpretation in the case of ***IIw*** is that the water added to the sample for dialysis suddenly converts all remaining TBOZ into zirconia, just as with ***Iw***. However, since there remains only a low amount of TBOZ after 4–7 days of condensation time in ethanol, and it is mostly chemisorbed at the surface, this process does not connect the particles chemically, and the aggregation is not significant. The basicity of the dialyzing water may influence the relative rates of the hydrolysis and condensation of TBOZ, which determines whether the deposition of TBOZ is mostly taking place on the surface or next to the NPs. The basic pH might be slowing down hydrolysis and speeding up condensation, as a result of which zirconia is deposited on the existing surface and not between the NPs, thus reducing aggregation of the NPs. The spontaneous redispersion means that the formed aggregates are in a metastable state that can be solubilized again by Brownian motion even at 4–8 °C.

Colloidal stability also depended on the size of the NPs: smaller NPs (***SZ3-4***) were more prone to aggregation than larger ones (***SZ1-2***) (Figure S5 in Supplementary Materials). Due to their higher surface-to-volume ratio, smaller NPs can adsorb a larger amount of TBOZ, which more easily connects the particles upon dialysis when it suddenly is converted into zirconia.

It is also important to note here that the pH outside of the dialysis membrane always remained higher than the pH inside the membrane, and both values were continuously decreasing in time. Using ultra-pure water without pH control resulted in a sample pH of 5.5–5.8. When basified water was used, the pH of the final sol varied between 6.9–7.7. The reason for the aqueous sol becoming more acidic with increasing aging time may either be the continued dissolution of the silica core through the porous zirconia shell (porosity investigated in paragraph 3.5) or processes altering the composition of the surface attached carbonate species. In any case, it was not possible to completely prevent the aggregation of the native NPs by further increasing the pH of the dialyzing water (10.4), at which point the dissolution of silica would become dominant.

When dialysis was carried out on native NPs aged for 3 weeks after deposition (***IIIw***), the sol did not whiten during dialysis. However, fewer primary particles were observed by DLS (Figure S5 in Supplementary Materials) than for the samples dialyzed between 4–7 days. According to these results, it was disadvantageous to wait 3 weeks before transferring the NPs into water.

Besides the effect on sol dispersity, we studied the structural changes induced by the transfer of the NPs from ethanol into the water by TEM, XRD, NMR, FTIR and zeta-potential measurements.

**Figure 2:**
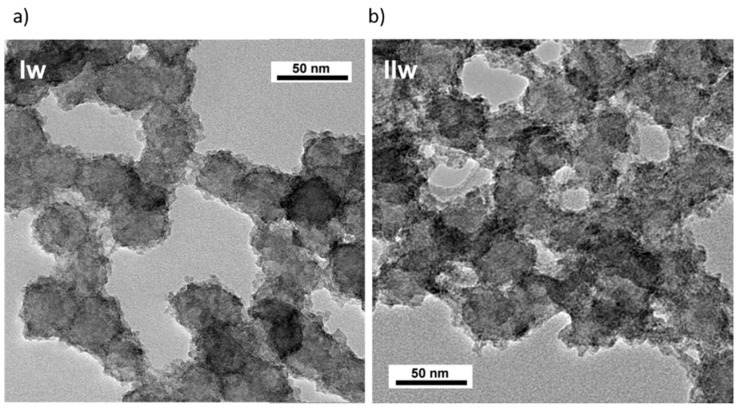
TEM pictures of ***SZ3*** (50 nm diameter) NPs after dialysis into water at different age: (**a**) ***Iw*** at 1. day) and (**b**) ***IIw*** at 7. days. Both samples presented rough NP surface, showing a structural change of zirconia when transferred from ethanol to water.

Structural changes were seen in TEM pictures of the native NPs upon transfer into the water at either 1 or 4 days of age (Figure 2): the surface of the NPs became rough, being covered by zirconia crystallites, also presenting spurs. For ***Iw*** and ***IIw***, we observed zirconia crystallites exclusively at the surface of silica cores, not next to them.

The X-ray diffractogram of ***Iw*** indicated a small crystallite size similar to that of the ethanolic sol. According to the Rietveld refinement, more than half of the formed crystallites were in the cubic phase (55.1%), accompanied by the monoclinic phase (41.2%) and the newly arisen tetragonal phase (3.7%) (Figure S6 in Supplementary Materials). This was the highest cubic phase ratio observed in our study.

**Figure 3:**
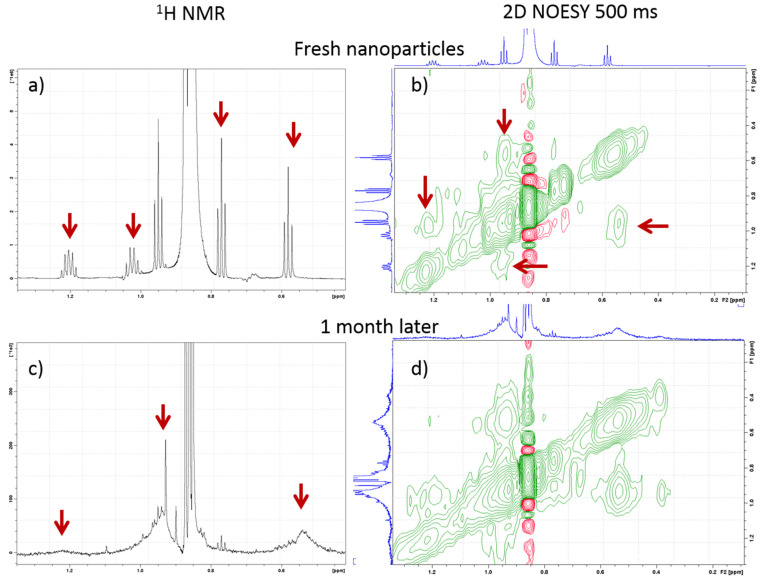
One-dimensional ^1^H NMR spectrum of fresh NPs of ***IIIw*** showing narrow signals of TBOZ (**a**), between which negative NOE cross peaks are observed in 2D NOESY spectra (**b**). One month later broad signals of residual TBOZ are detected (**c**), which also showed negative NOE cross peaks (**d**).

To confirm our hypothesis on physisorbed TBOZ driving the aggregation behavior of the NPs transferred into the water, we investigated residual TBOZ signals in the NMR spectra of the dialyzed samples. Free and surface-bound TBOZ species were observed in NMR spectra at any age of the sample after dialysis into water. ^1^H NMR spectra of the ***IIIw*** featured multiplets in the aliphatic region (Figure 3a), which were assigned to residual methyl groups of tetrabutyl moieties of TBOZ. The 2D nuclear Overhauser effect spectroscopy (NOESY) spectra showed negative NOE cross-peaks between these signals (Figure 3b). The narrow resonances in the TBOZ multiplets became broad after a month spent in water (Figure 3c) while still showing negative NOE cross-peaks between them (Figure 3d). The initial narrow resonances with negative NOEs indicate the attachment of loosely associated TBOZ on the NP surface with a fast exchange of the TBOZ molecules between surface-bound and solution-state free forms. Broadening of the signals later suggests stronger attachment to the zirconia surface, indicating chemisorption of the rest of TBOZ to the zirconia surface in the aqueous environment.

**Figure 4:**
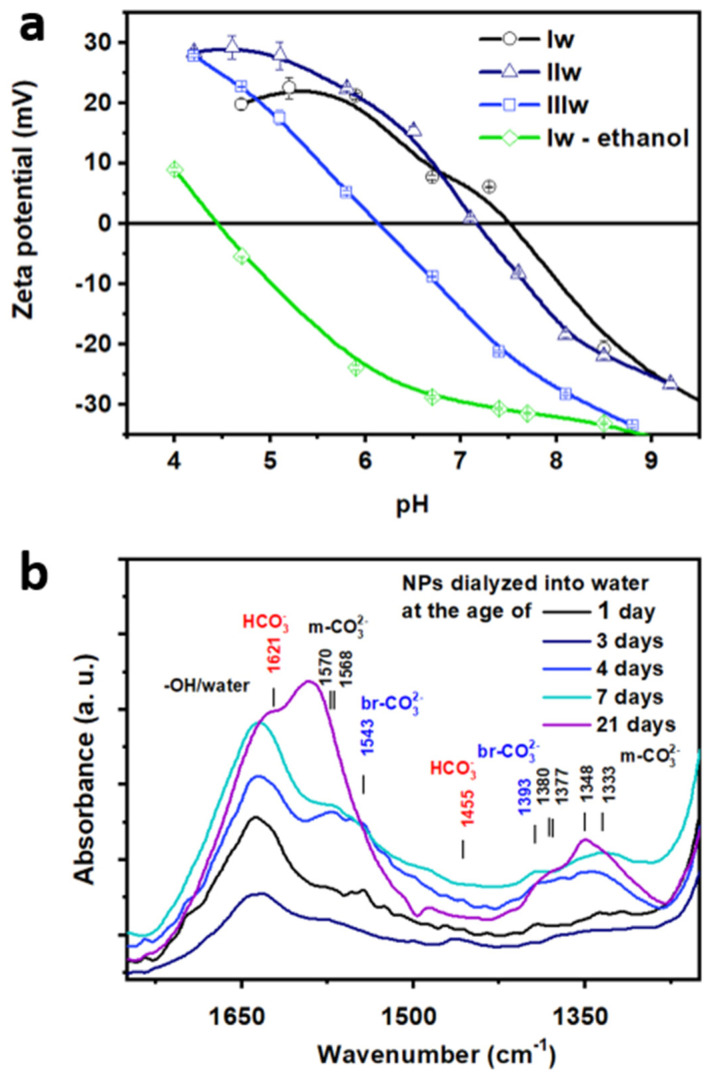
Zeta potential vs. pH curves (**a**) and FTIR spectra (**b**) and of ***SZ3*** (50 nm diameter) NPs after dialysis into the water at different ages: ***Iw*** (1 day), ***IIw*** (7 days), ***IIIw*** (21 days) and ***Iw-ethanol*** (freshly dialyzed against ethanol and then against water). The zeta potential measurements show that the IEP of zirconia is continuously decreasing with time, which will affect the colloidal stability and the adsorption properties of the nanocarrier. The FTIR results reveal that the difference in the surface chemistry of samples transferred into water at different ages is significant.

The IEP of the NPs as determined by zeta potential vs. pH curves shown in Figure 4a was monotonously decreasing with the age of the transferred sol from 7.4 (***Iw***) through 7.2 (***IIw***) to 6.1 (***IIIw***). Thus, depending on condensation time, the native NPs are stabilized by electrostatic repulsion between negative charges at higher, alkaline pH, prone to aggregation at intermediate values and could, in theory, be again stable at acidic pH by repulsion between positive surface charges. However, increased silica dissolution prevents the application of acidic pH for practical purposes.

As a final proof for our hypothesis of TBOZ physisorption on the zirconia surface after deposition, we carried out an experiment whereby the freshly prepared ***SZ3*** was first dialyzed against ethanol, and then the dialysis was continued against basic water (***Iw-ethanol***, green line in Figure 4b). In this case, the IEP (4.4) was strikingly lower compared to all the other samples described above. It was closer to that of native silica surface (~2) [38] and was much lower than the IEP of native pure zirconia surface (8.2–observed on similarly prepared pure zirconia particles [39]). We think that the physisorbed TBOZ was removed during the ethanolic dialysis leaving a thin layer of chemisorbed zirconia on the surface, which only partially covers the silica core reflected by the IEP closer to that of silica instead of zirconia. This finding indicated the reversibility of the surface modification on the day of the deposition reaction and supports our hypotheses on the temporary formation of a physisorbed TBOZ multilayer.

The FTIR study of the dialyzed native NPs revealed an alteration of the silica nanoparticulate structure for samples dialyzed without temperature or pH control. As an example of native NPs dialyzed into the water without temperature control (Figure S7 in Supplementary Materials) and an example of surface-modified samples dialyzed without pH control (Figure S8 in Supplementary Materials). The figures show that the intensity of the νSi-O-Si vibrational band at 1090 cm^−1^ is drastically reduced. This is probably a result of silica dissolution that has been reported and studied by several research groups for mesoporous silica in simulated body fluid [40,41,42] but has also been reported to occur in neutral-alkaline water [43]. For ***SZ4,*** with the smallest particle size, the zirconia shell did not remain attached to the core after dialysis even under controlled conditions, as indicated by the reappearance of vibrational bands of native silica surface in the FTIR spectrum. Thus, ***SZ3*** was found to be the smallest suitable particle size, and we conducted all further studies on this sample.

We found significant differences in the surface chemistry of the aqueous samples according to the age of ***SZ3*** at the start of the dialysis using FTIR spectroscopy (Figure 4b). If the dialysis was carried out in the first 3 days after zirconia deposition (***Iw***), the hydrogenocarbonate species (1621 and 1445 cm^−1^) were predominant next to the vibrational bands of monodentate carbonate (1570, 1568, 1380, 1377, 1348 and 1333 cm^−1^). Bridged carbonate was visible first on the surface of the sample dialyzed on the third day (1543 and 1393 cm^−1^). The samples transferred at the age of 4–7 days (***IIw***) showed a surface with a similarly high amount of monodentate and bridged carbonates. For samples transferred at the age of 20 days or more (***IIIw***), the monodentate form was predominant. The trend observed for the ripening of the zirconia shell in ethanolic sol, as described with respect to Figure S4, are thus conserved after transfering the NPs into water: the more acidic hydrogenocarbonate species are gradually replaced by the alkaline monodentate and bridged carbonate as zirconia is further aged in the ethanolic solution. This is in agreement with observation of increasing acidification of the sols with time. The carbonate species could contribute to this process by two consecutive processes that also cause acidification: (a) protons could be removed from surface in the process of hydrogenocarbonate to monodentate carbonate conversion, and (b) monodentate carbonates could become bridged carbonates releasing a CO_2_ molecule into the solution, which can further acidify the medium. These differences in the surface chemistry of the zirconia show the importance of the careful timing during the synthesis, as the resulting material surface is highly dependent on it.

### 3.3. Aging of Native Silica@zirconia Core@shell NPs in Water Following Dialysis

As the NMR study suggested, the transformation of the NP surface did not stop after dialyzing the NPs into water, which is why we carried out a follow-up study using FTIR on samples ***Iw***, ***IIw*** and ***IIIw*** in the weeks following the dialysis.

The enlarged region of hydrogenocarbonate/carbonate vibrational bands showed that the surface structure did not change in the first week in the ***Iw*** sample, and then the carbonate vibrational bands (1544, 1465 and 1389 cm^−1^) slowly disappeared (Figure S9 in Supplementary Materials). For ***IIw***, the sample aged for 7 days before transfer into the water, we observed a high amount of hydrogenocarbonate and m-CO_3_^2−^/br-CO_3_^2−^ on the surface, which were still partly present 5 weeks after dialysis with an increased ratio of bridged carbonate. ***IIIw*** had a lower amount of binding carbonate species after 3 weeks of time spent in water than ***IIw*** after 5 weeks in water, though the composition of surface species was similar. ***IIw*** and ***IIIw*** samples kept their colloidal stability for at least two months. The longest persistence of surface carbonate species that indicates the highest binding capacity of the zirconia surface was observed for ***IIw***.

In conclusion, we determined that the optimum time for transferring the NPs into the water was 4–7 days of ripening after synthesis because not only the NP surface is best covered by accessible sites, but also the dispersity and surface properties of the samples are stable for the longest time, at least for 5 weeks. We also established that the native NPs are best stabilized depending on the aging time at neutral-alkaline pH, whereby electrostatic repulsion between negatively charged surface prevent aggregation.

### 3.4. Adsorption of Deoxynucleoside Monophosphates on Silica@zirconia NPs

As we observed a significant transformation of the zirconia shell during the transfer of the core@shell NPs into water, we expected to see differences in their adsorption properties as well. We undertook investigations to identify the experimental design that leads to the maximum surface loading and stability of the vaccine nanocarriers. Two experimental designs were used for the surface modification of the NPs differing in the order of steps: (i) NP synthesis-dNMP functionalization-dialysis, and (ii) NP synthesis-dialysis-dNMP functionalization-dialysis. In the first method, an aqueous solution of dNMPs was added to the NPs of different ages (0, 6 and 21 days of condensation time) in ethanol, followed by the dialysis of the NPs into the water. In the second design, the dialysis of the NPs took place first (after 4 days of condensation time), and then the aqueous dNMP solution was added and followed by further dialysis. We refer to these two methods as the ethanolic and the aqueous surface modification methods.

**Figure 5:**
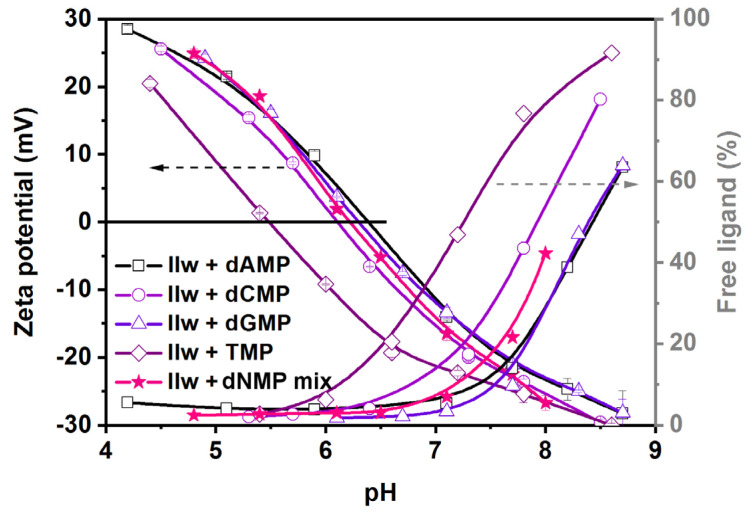
Variation of zeta potential (**left axes**) and the percentage of free ligands (**right axes**) in the function of pH for single-component dNMP solutions and a four-component dNMP mixture. The resulting IEP was 6.3 ± 0.2 for all, except for TMP ligand, whose adsorption took place at lower pH, below pH 7. For the three other single components and the dNMP mixture, the adsorption was almost complete at pH below 7.5.

In the first study, we identified the optimal pH range for the adsorption of single dNMP solutions and four-component mixtures. For this, we used the aqueous surface modification method, adding 63 mg ligands to 1 mg of NPs (without the last dialysis step) and performed a pH-dependent zeta potential study. We recorded the free ligand content of the supernatant by UV detection at the same time after centrifugation of the NPs at each pH (Figure 5).

Specific adsorption took place in the acidic-neutral pH range in all of the examined cases of single- and four-component mixtures, as evidenced by the low free ligand content in the supernatant (Figure 5). In general, in the acidic pH range, the surface of NPs was somewhat positive upon surface modification, which was gradually turning to neutral around the IEP (pH 6.3 ± 0.2) and further to strong negative charges as the pH was increased accompanied by the desorption of the ligands. As the electrostatic repulsion, either between positive or negative charges, is a strong determinant of colloidal stability, it was extremely important to control the pH of the surface-modified NPs. We demonstrated that at neutral-acidic pH, the silica cores started to dissolve (especially at room temperature), and the present results indicate that in this region, they are also prone to aggregation due to low surface charge. On the other hand, high surface loading can only be reached at a pH lower than 7.5. This delimits a narrow useful pH range for the efficient surface modification and the use of silica@zirconia NPs with dNMPs to pH 7.0–7.5. This is not completely in line with the observations of Wu et al., who reported a pH range of 5–7 as optimal for DNA adsorption on titania/zirconia NPs [44]. Although the stability of the nanocarriers is just as good at pH 5 (due to a positively charged surface) as at pH 7, we found that the addition of single components or a dNMP mixture solution at the native pH (pH 4.9) yielded aggregated samples. Since the target nucleic acid sequences contain all nucleotides, we continued the optimization of the surface modification using the dNMP mixture as the ligand solution in the pH range of 7.0–7.5.

### 3.5. Surface Modification in Ethanol vs. in Water and the Effect of Zirconia Shell Ageing

We compared the ethanolic and aqueous surface modification methods using a dNMP mixture ligand solution at a controlled pH and ***SZ3*** NPs of 50 nm mean diameter (Table 3). Since the NPs are colloidally more stable above pH 7 due to electrostatic repulsion between negative surface charges, while the ligand adsorption is favored at acidic pH, the pH used in this study for the NPs and the dNMP mixture was chosen to be pH 6.0 to aid swift ligand adsorption, and the resulting solution was dialyzed against alkaline water to prevent aggregation. Three samples were surface modified using the ethanolic procedure at the age of 0, 6 and 20 days after dialysis (***INu***, ***IINu*** and ***IIINu***, respectively), and one sample was surface-modified using the aqueous procedure (***IIwNu***), which was dialyzed into the water 7 days after synthesis, which was found optimal in the previous investigations described in paragraph 3.2. We investigated the pH of the samples, the dispersity with DLS, the structure and composition of the surface with FTIR and the morphology of the NPs by TEM, and we determined the loading capacity of the particles by quantitative analysis with TGA and desorptional titration.

Similarly, to the case of native silica@zirconia NPs, we observed that independently of the medium of the surface modification and despite the careful pH control of the dialyzing water, the pH of the resulting samples was gradually decreasing with age, e.g., 6-week-old ***INu*** had a pH of 6.7 in contrast to pH 8.2 when freshly made (Table 3). We ascribe this phenomenon to the continuous dissolution of the silica core due to the microporous structure of the zirconia layer (see below), which was not inhibited by the surface modification. Thus, the pH of the sample ought to be controlled for longer-term stability.

**Figure 6:**
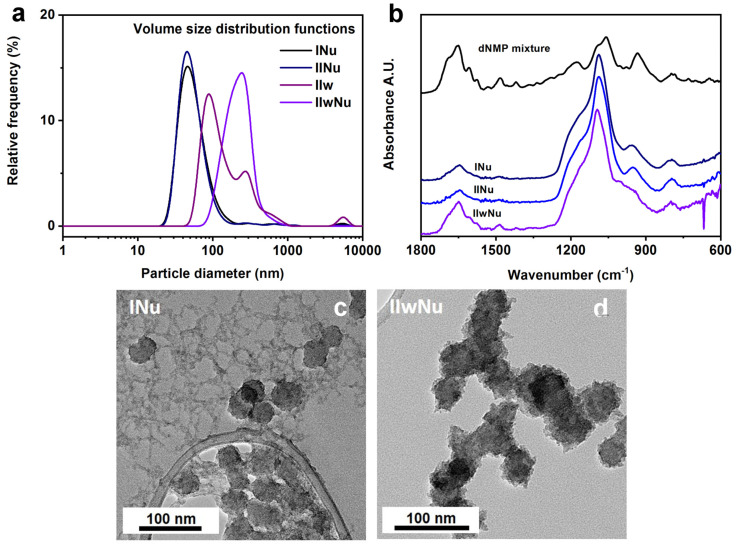
A comparison of samples surface modified in ethanol and in water by (**a**) DLS, (**b**) FTIR spectra and (**c**–**d**) TEM pictures. The dispersity of the sol is affected by the ethanolic and the aqueous surface modification methods (**a**). A higher surface load can be achieved by surface modification in water (**b**). The addition of aqueous ligand solutions always results in the deposition of zirconia next to the NPs using the ethanolic surface modification method, as shown for dNMP mixture in (**c**) for sample ***INu***, while this is not observed for surface modification after transfer into water (**d**) for sample ***IIwNu***.

Despite the highly charged surface for samples ***INu***, ***IINu*** and ***IIwNu*** (Table 3), the dispersity of the resulting samples could not be completely restored after solvent exchange according to DLS. While large aggregates were present in very low amounts for the samples surface modified using the ethanolic procedure at 0 or 6 days of shell condensation (***INu, IINu)***, there were smaller clusters of primary particles in a higher amount for samples surface modified with the aqueous procedure (***IIwNu)*** (Figure 6a). The sample surface modified using the ethanolic procedure at 20 days of shell condensation (***IIINu)*** was found to be strongly aggregated.

The silica nanoparticulate structure was preserved during both surface modification processes according to the FTIR spectra. When spectra were normalized for the νSi-O-Si band at 1080 cm^−1^, it became clear that the aqueous surface modification led to higher dNMP cargo load, as indicated by the higher intensity of vibrational bands at 1650 cm^−1^, 1607 cm^−1^, 1486 cm^−1^, and 1575 cm^−1^ (Figure 6b).

TEM pictures revealed that during the surface modification in ethanol, part of the zirconia deposited next to the NPs, giving rise to an inhomogeneous system (Figure 6c). When the surface modification was carried out after the transfer of NPs into the water, zirconia was only deposited on the silica surface and did not form stand-alone crystallites (see Figure 6d). This analysis showed that the addition of the aqueous ligand solution to the NPs in ethanol also provoked a sudden crystallization of the unreacted TBOZ, but the TBOZ deposited next to the particles, unlike during the dialysis of native NPs when the condensation of the unreacted TBOZ caused the aggregation. This observation was identical in all cases of single-component ligand solutions and the four-component mixture. We hypothesize that the ligands quickly exchange the surface-adsorbed TBOZ; thus, the organometallic compound cannot deposit at the surface of NPs anymore but reacts readily with the injected water forming stand-alone ZrO_2_ crystallites. However, in the case of the aqueous surface modification, the unreacted TBOZ physisorbed on the NPs are already removed by the preceding dialysis step when the ligand solution is added to it, so no zirconia aggregates are formed. Altogether, these results demonstrated the clear superiority of the aqueous surface modification method.

Quantitative analysis of the bound dNMP mixture was performed by desorption titration on sol samples as well as by TGA on dried powders (Table 3). In order to evaluate the quantity of dNMP mixture desorbed from the surface of NPs at pH 8.8, the UV-visible absorption of the supernatant was evaluated at this pH based on a calibration curve and the solid content of the samples (Figure S10 in Supplementary Materials). There was a good agreement between the quantities obtained by the two methods (except for ***INu***) and a clear trend was found: although the adsorption of ligands was observed to become complete at an acidic pH, below 6.5 or lower, the capacity of NPs to bind ligands was decreasing with growing age despite the decreasing pH. According to XRD and FTIR investigations on the transformation of crystalline phases on the NP surface, as described in Section 3.1, the decreasing binding capacity is probably due to the ripening of the zirconia shell accompanied by a reduction of the number of crystal lattice defects. The surface load was found to be the highest (207 mg/g) for sample ***IIwNu*** surface modified in water following dialysis on day 6 of the shell condensation. This capacity is much higher than the value reported by Wu et al. [44], capturing nucleosides from urine with titania-zirconia NPs (35 mg/g). The material synthesized by Wu et al. is similar in composition to our system, but they used surfactant-assisted deposition of mesoporous titania-zirconia coating on the surface of mesoporous silica and had to eliminate surfactants at 400 °C. This had obviously reduced the adsorption capacity of the obtained material even though they had achieved a high surface area (350 m^2^/g).

The BET-specific surface area of dried ***SZ3*** was assessed by the N_2_ adsorption-desorption method and was found to be 315 m^2^/g, which is somewhat smaller obtained by Wu et al. and higher compared to the surface load. Some mesoporosity was found in ***SZ3***, but this we attributed to interparticulate spaces (Figure S11 in Supplementary Materials). The microporosity of the sample was determined to be 0.121 cm^3^/g with a mean pore diameter of 1.4 nm. Thus, the silica@zirconia NPs prepared under mild wet conditions in our work presented similar surface area, with more than 5 times the adsorption capacity of the mesoporous titania-zirconia described by Wu et al. The research group of Badruddoza [45] captured nucleosides using cyclodextrin-modified magnetic NPs, whereby the binding mode was based on Van der Waals and hydrophobic interactions, which is thus different than the electrostatic interaction-based adsorption in our system. They achieved a load of 12.4 mg/g (adenosine) and 29.9 mg/g (guanosine), which is also considerably lower than obtained in our study.

To summarize, we found that the optimal way to prepare highly loaded silica@zirconia materials is to use the smallest stable size (50 nm in our study), transfer the NPs into cold, basic water after an ideal aging period of 4–7 days and carry out surface modification in water with a dNMP mixture at a controlled pH (7.0–7.5). The regulation of the pH plays a crucial role in the long-term colloidal stability of the sample; therefore, the possibility of buffering was further investigated.

### 3.6. Influence of Buffers on the Adsorption Equilibrium

In view of further biological applications, we selected several buffers based on the suggestions of Ferreira et al. [31] and studied the adsorption of the dNMP mixture on the surface of the NPs dialyzed into the water after 6 days of shell condensation (sample ***IIw***), in the presence of 10 mM HEPES, PIPES, MES, MOPS, MOPSO and K-phosphate.

The study was conducted under identical conditions as described earlier in the investigation of the dNMP adsorption. The zeta potential of the NPs and the UV spectrum of the supernatants were recorded, and the latter was analyzed for free ligand content (Figure S12 in Supplementary Materials). The zeta potential vs. pH curves of the dNMP-modified NPs was similar in the presence of PIPES, MOPS, MOPSO and MES and was shifted towards the acidic region compared to the non-buffered system without buffer (IEP 5.8 vs. IEP 6.3). This indicates the presence of stronger negative charges on the surface at the pH range of interest, between pH 7.0–7.5, in these buffers. In the presence of HEPES, we observed a more pronounced shift of IEP (pH 5.5). The percentage of free ligands showed a similar picture: it fell below 10% at pH 6.5 and lower for all the buffered systems except for phosphate buffer, for which it remained around 70%. K-phosphate buffer behaved completely differently from the other buffers, as the charge of the NPs was −31 ± 1 mV in the entire observed pH range, indicating a chemical transformation taking place on the surface. The phosphate ions compete with dNMP molecules and occupy most of the surface sites, thereby providing a high negative zeta potential in the whole of the observed pH range. As a result, 70% of the dNMP ligands remain free and cannot bind to the surface.

We concluded that the phosphate buffer was incompatible with our system. For the rest of the buffers, we did not see interference with the adsorption phenomenon. They are, therefore, all suitable buffers to control the pH and to ensure colloidal stability of the present system.

**Figure 7:**
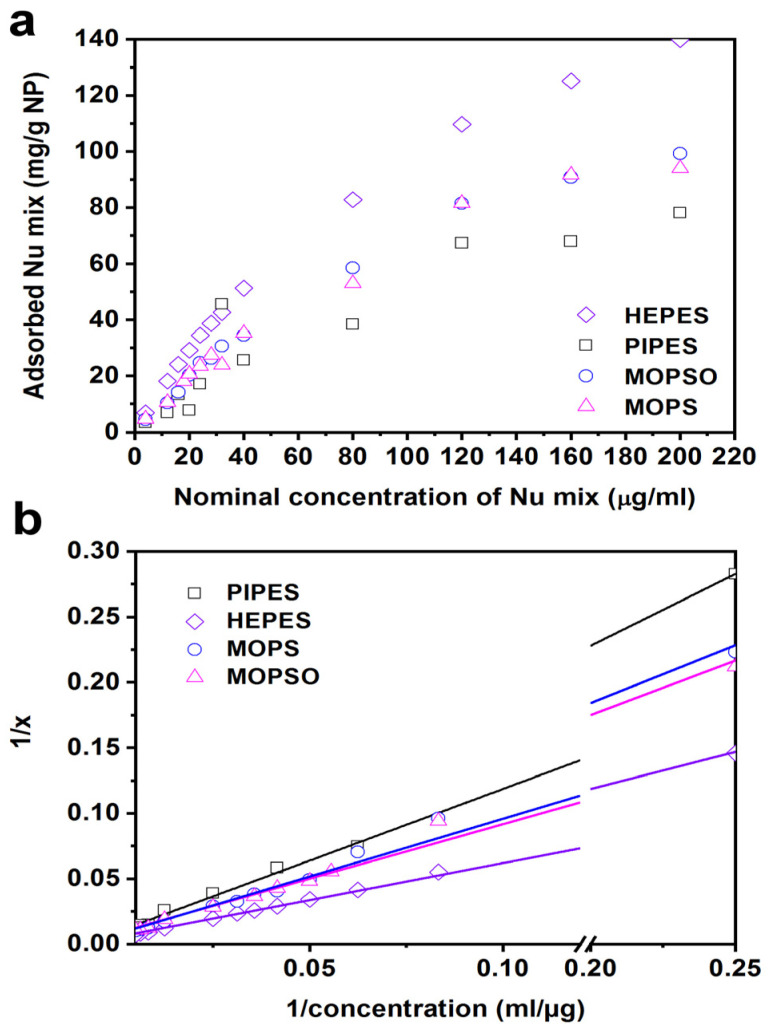
Adsorption isotherms of the dNMP mixture on ***IIw*** in 20 mM buffers at 22 °C (**a**) and a linearized plot used for fitting (**b**). The adsorption isotherms followed the Langmuir model, allowing us to derive the maximum adsorption capacity for each system.

Finally, in order to choose the best buffering medium that enables the highest amount of dNMP mixture adsorbed to the surface, we recorded adsorption isotherms in the four selected buffers (we discarded MES because of its upper buffering limit at pH 6.7) at 22 °C and pH 7.4 (Figure 7a). The isotherms followed the Langmuir model (Figure 7b); therefore, by plotting the reciprocal of the adsorbed amount of ligands against the reciprocal of the initial concentration, we obtained a linear relationship, which allowed us to evaluate the maximum adsorbing amount of ligands (q_m_) in each buffer (Table S1 in Supplementary Materials) [45,46]. The highest amount of dNMP mixture adsorbed to the surface was achieved for ***IIw*** in HEPES buffer: 187 mg/g (pH 7.4), which is also in agreement with its largest IEP shift corresponding to the highest negative charges on the surface. Comparing this value with the surface loading for non-buffered ***IIwNu obtained*** by TGA (207 mg/g; pH 6.9), we can state that the maximum load in a buffered system is only slightly lower than the one obtained in non-buffered one. We would expect that the maximum load may even be identical at pH 6.9.

In summary of these steps, we evidenced that silica@zirconia core@shell NPs prepared under mild wet synthetic conditions bind a high amount of dNMP on their surface and buffering necessary to prevent their early degradation does not inhibit this process. Moreover, the pH stability range of the nanocarriers coincides with the physiological pH optimal for the administration of vaccine delivery systems. This way, we confirmed that the buffered system sustained colloid stability and is suitable for its intended biological use.

## 4. Conclusions

Our paper presents the systematic progress towards the establishment of an optimized synthetic procedure of high adsorption capacity silica@zirconia nanocarrier.

We achieved high specific surface area nanocarriers through lowering particle size and using very mild synthetic conditions. We studied the time-dependent changes in the zirconia shell structure and optimized the solvent exchange and the surface modification accordingly. The aging time of the zirconia shell in the synthetic mixture largely determined the colloidal stability and the aggregation behavior of the NPs during the dialysis step due to a layer of physisorbed TBOZ zirconia starting material attached to the surface. Furthermore, the pH was found to be crucial for the maintenance of colloid stability during and following the surface modification, with the optimum being between pH 7.0–7.5. We tested several biocompatible buffers of which HEPES, PIPES, MOPS and MOPSO were proven to be suitable and compatible with our nanocarrier system. Based on these results, we anticipate stable and high-capacity adsorption of nucleic acid-type antigens or adjuvants on the surface of the optimized NPs, and in our future work, we proceed to the elaboration of vaccine adjuvant nanocarriers using these cargo molecules.

## Figures and Tables

**Table 1:** The mean size and size distribution of NPs.

Sample	SiO_2_ Core TEM Mean Ø * (nm)	SiO_2_ Core DLS Volume Mean ** (nm)	SiO_2_@ZrO_2_ TEM Mean Ø * (nm)	SiO_2_@ZrO_2_ DLS Volume Mean ** (nm)	SiO_2_@ZrO_2_ DLS PdI
***SZ1***	81 ± 12	123 ± 34	95 ± 12	109 ± 29	0.021
***SZ2***	55 ± 8	75 ± 25	63 ± 6	134 ± 53	0.133
***SZ3***	40 ± 6	35 ± 13	48 ± 5	47 ± 13	0.043
***SZ4***	13 ± 2	6 ± 3	20 ± 3	25 ± 6	0.021

* TEM diameter evaluated on 50–100 NPs, ** hydrodynamic diameter, PdI: polydispersity index.

**Table 2:** The proportion of zirconia crystalline phases in core@shell NPs as a function of age.

Sample	Fm-3m (Cubic)	P121/c1 (Monoclinic)
***SZ3* fresh**	0.04%	99.96%
***SZ3* 1 week**	36.08%	63.92%
***SZ3* 2 weeks**	22.02%	77.98%
***SZ3* 1 year**	14.33%	85.67%

**Table 3:** A comparison of stability and dNMP content of NPs surface modified in ethanol and in water. ***INu***, ***IINu*** and ***IIINu*** are samples modified using the ethanolic procedure at the age of 0, 6 and 20 days after dialysis, and the ***IIwNu*** sample is treated by the aqueous procedure, which is dialyzed into water 7 days after synthesis.

Sample	pH at the End of Dialysis	Zeta Potential (mV)	Z-Average (nm) and PdI from DLS	dNMP Mix Content from Titration		dNMP Mix Content from TGA	
				mg/g	µmol/m^2^ *	mg/g	µmol/m^2^ *
***INu***	8.2	−36 ± 2	106 (0.586)	196	1.26	124	0.74
***IINu***	7.4	−39 ± 1	107 (0.501)	108	0.63	120	0.71
***IIINu***	5.7	−3 ± 1	high	53	0.29	63	0.33
***IIwNu***	6.9	−30 ± 1	247 (0.179)	n.a.	n.a.	207	1.34

* Calculated using the volume average mean diameter of the sample. PdI: polydispersity Index.

## Supplementary Materials

The following are available online at https://www.mdpi.com/article/10.3390/nano11092166/s1, **Figure S1:** TEM pictures of silica core for batches ***S1***, ***S2***, ***S3*** and ***S4*** and silica@zirconia core@shell for batches ***SZ1***, ***SZ2***, ***SZ3*** and ***SZ4*** NPs, **Figure S2:** DLS size distribution functions of core and core@shell NPs, **Figure S3:** XRD spectrum of 2-week-old SZ3 NPs, line fitted using Rietveld analysis and the error to the fit, **Figure S4:** FTIR spectra of **SZ3** silica@zirconia core@shell NPs in ethanol at different aging times after deposition, **Figure S5:** DLS size distribution functions of different size NPs dialyzed into water at pH 9 at the age of 4-7 days after deposition of zirconia shell and 50 nm diameter particles dialyzed after different aging times at pH 9, **Figure S6****:** X-ray diffractogram and Rietveld refinement evaluation of ***Iw*** powder, **Figure S7****:** FTIR spectra of native NPs dialyzed into basic water without temperature control, **Figure S8****:** FTIR spectra of ***SZ1*** NPs surface modified in ethanol without controlling the pH of ligand solutions and ***SZ3*** NPs surface modified freshly after synthesis in ethanol using dNMP mixtures at pH 6.0, **Figure S9:** Structural changes of ***SZ3*** core@shell NPs after transfer into water by dialysis at pH 9 at different ages according to FTIR spectra. ***Iw*** (transfer on day 1 after zirconia deposition); ***IIw*** (transfer on day 7); ***IIIw*** (transfer on day 21), **Figure S10:** Combined zeta potential and free ligand quantity of the supernatant vs. pH curves obtained during desorption titration and calibration curve used for ***Nu*** mix quantity assessment, **Figure S11****:** N_2_ adsorption-desorption isotherm and micropore size distribution of ***SZ3*** (dried at the age of 4 days) powder, **Figure S12:** Variation of the zeta potential (left axis) and the percentage of free ligands (right axis) in the function of the pH for the dNMP mixture in the presence of 10 mM buffers, **Table S1:** Goodness of fit and maximum absorbing dNMP mixture on ***IIw*** in 20 mM buffers at 22 °C and at pH 7.2.
