# Supplementary material for: Silica@zirconia Core@shell Nanoparticles for Nucleic Acid Building Block Sorption
Source: Nanomaterials (Basel). 2021 Aug 25;11(9):2166. doi: 10.3390/nano11092166 (PMC8468278; doi:10.3390/nano11092166)
Supplement: Supplementary file 1 [file nanomaterials-11-02166-s001.zip › nanomaterials-1303495-supplementary.pdf]

nanomaterials

Supplementary Materials

Silica@zirconia Core@shell Nanoparticles for Nucleic Acid Building Block Sorption

Livia Naszályi Nagy [1], Evert Dhaene [2], Matthias Van Zele [2], Judith Mihály [3], Szilvia Klébert [3], Zoltán Varga [3], Katalin E. Kövér [4], Klaartje De Buysser [2], Isabel Van Driessche [2], José C. Martins [1] and Krisztina Fehér [5,*]

[1] NMR and Structure Analysis Research Group, Department of Organic and Macromolecular Chemistry, Ghent University, Krijgslaan 281 S4, B-9000 Ghent, Belgium; lnaszalyi@gmail.com (L.N.N.), jose.martins@ugent.be (J.C.M.)
[2] Sol-Gel Centre for Research on Inorganic Powders and Thin Films Synthesis, Department of Chemistry, Ghent University, Krijgslaan 281 S3, B-9000 Ghent, Belgium; Evert.Dhaene@UGent.be (E.D.); Matthias.VanZele@ugent.be (M.V.Z.); Klaartje.DeBuysser@ugent.be (K.D.B.); Isabel.VanDriessche@ugent.be (I.V.D.)
[3] Institute of Materials and Environmental Chemistry, Research Centre for Natural Sciences, Eötvös Loránd Research Network (IMEC RCNS ELKH), Magyar tudósok körútja 2, H-1117 Budapest, Hungary; mihaly.judith@ttk. hu (J.M.); klebert.szilvia@ttk.hu (S.K.); varga.zoltan@ttk. hu (Z.V.)
[4] Department of Inorganic and Analytical Chemistry, University of Debrecen, Egyetem tér 1., H-4032 Debrecen, Hungary; kover@science.unideb.hu
[5] Molecular Recognition and Interaction Research Group, Hungarian Academy of Sciences-Eötvös Loránd Research Network at University of Debrecen, Egyetem tér 1, H-4032 Debrecen, Hungary
* Correspondence: feher.krisztina@science.unideb.hu; Tel.: +36-52-512-900

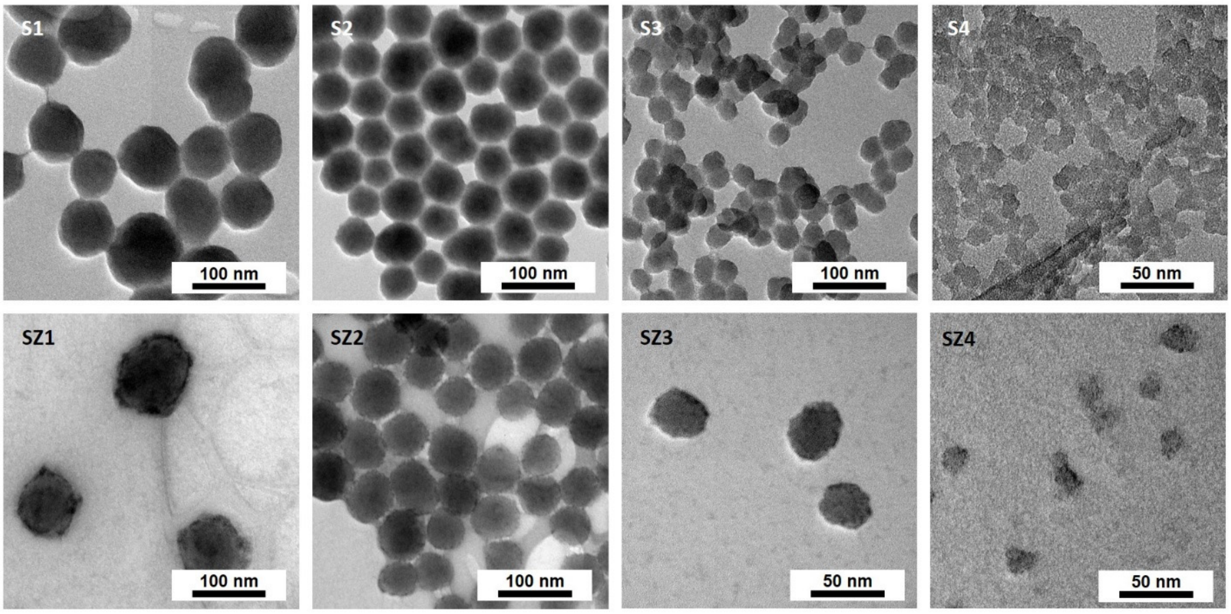

Figure S1. TEM pictures of silica core for batches ***S1***, ***S2***, ***S3*** and ***S4*** (upper row) and silica@zirconia core@shell for batches ***SZ1***, ***SZ2***, ***SZ3*** and ***SZ4*** (row below) NPs.

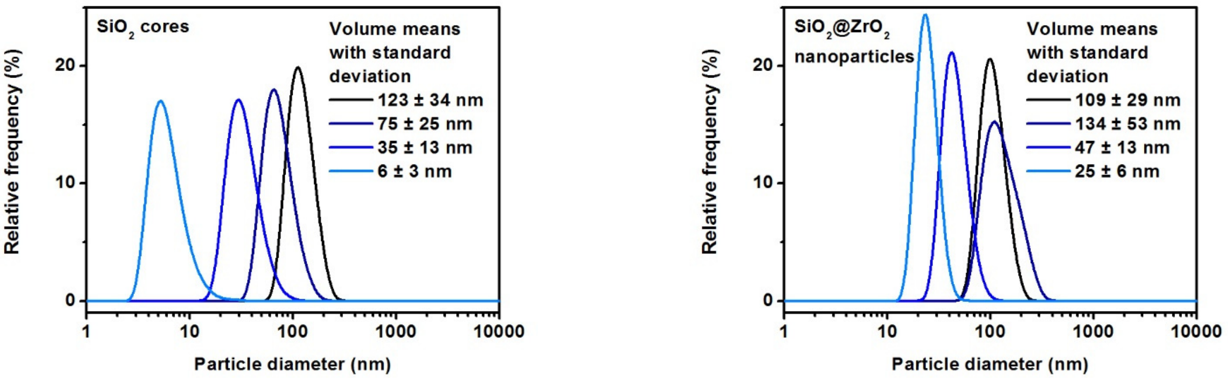

Figure S2. DLS size distribution functions of core (left) and core@shell (right) NPs.

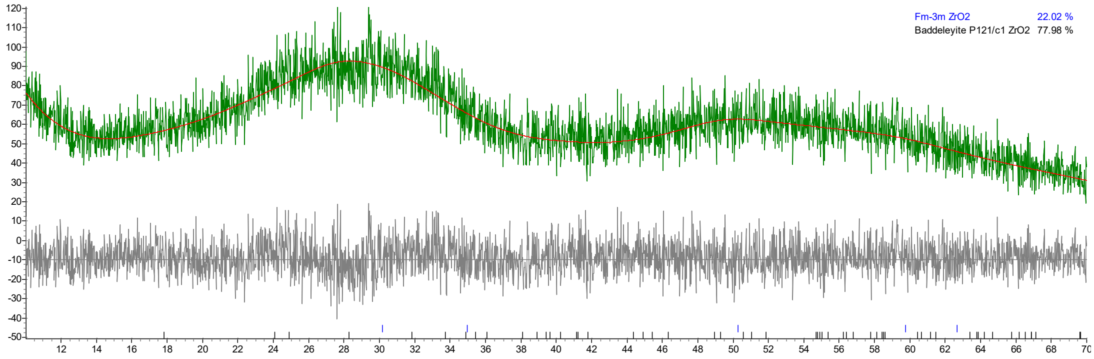

Figure S3. XRD spectrum of 2-week-old SZ3 NPs (green line), line fitted using Rietveld analysis (red line) and the error to the fit (grey line).

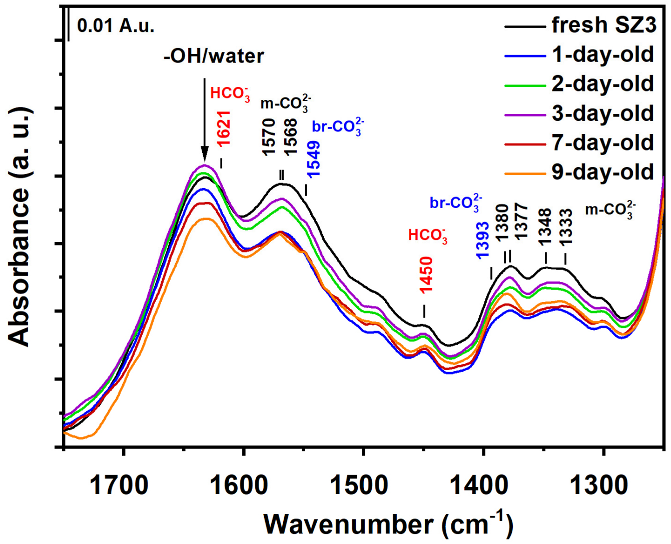

Figure S4. FTIR spectra of **SZ3** silica@zirconia core@shell NPs in ethanol at different aging times after deposition.

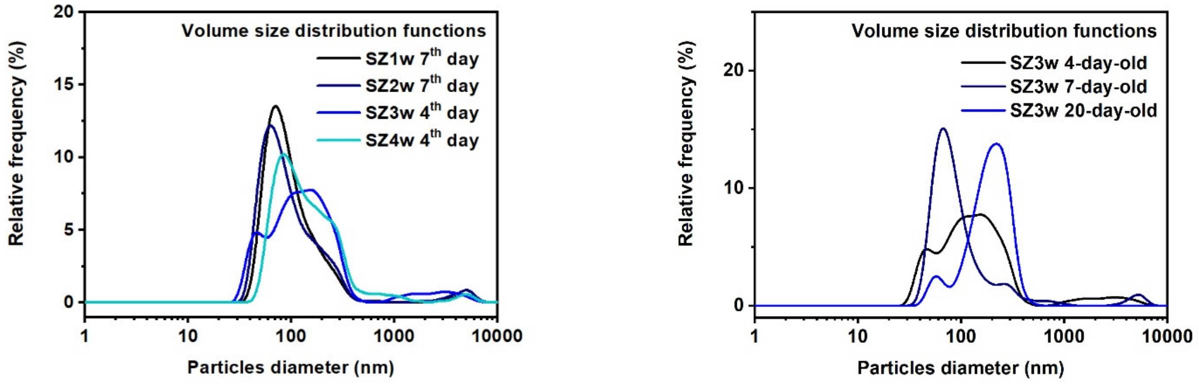

Figure S5. DLS size distribution functions of different size NPs dialyzed into water at pH 9 at the age of 4-7 days after deposition of zirconia shell (left) and 50 nm diameter particles dialyzed after different aging times at pH 9 (right).

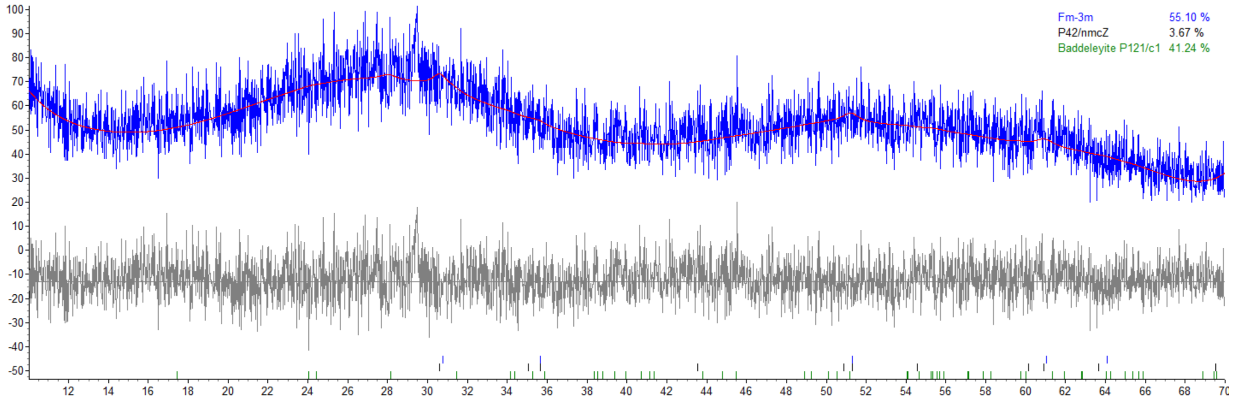

Figure S6. X-ray diffractogram and Rietveld refinement evaluation of ***Iw*** powder.

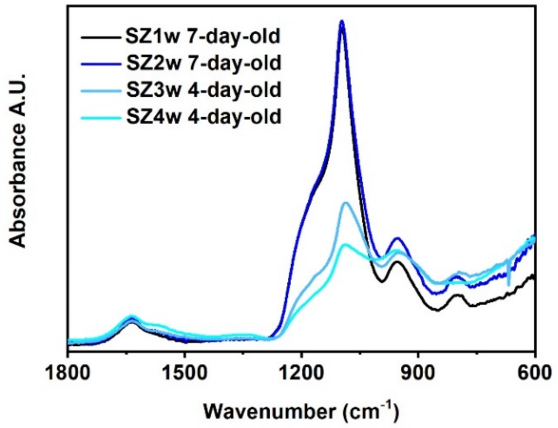

Figure S7. FTIR spectra of native NPs dialyzed into basic water without temperature control.

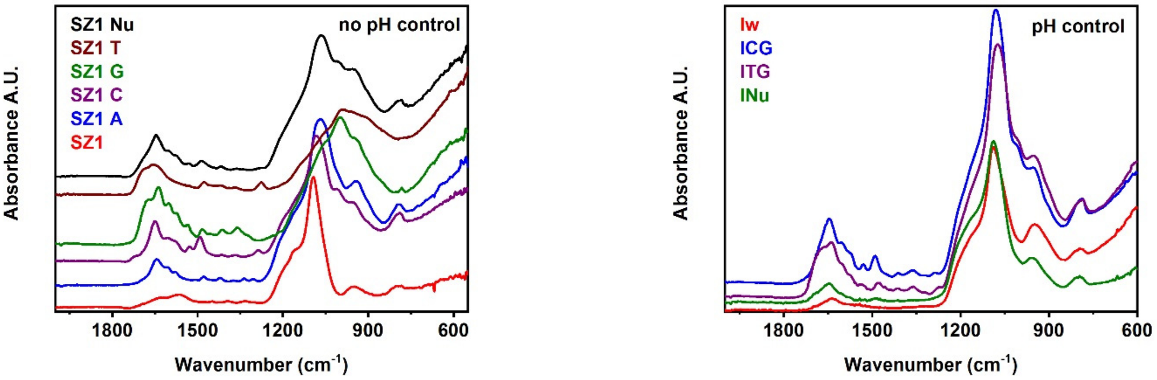

Figure S8. FTIR spectra of ***SZ1*** NPs surface modified in ethanol without controlling the pH of ligand solutions (left) and ***SZ3*** NPs surface modified freshly after synthesis in ethanol using dNMP mixtures at pH 6.0 (right). ***Nu*** stands for dNMP mixture, ***T*** stands for TMP, ***G*** stands for dGMP, ***C*** stands for dCMP, ***A*** stands for dAMP, ***CG*** stands for binary dCMP-dGMP mixture, ***TG*** stands for binary TMP-dGMP mixture. All these samples were surface modified in ethanol.

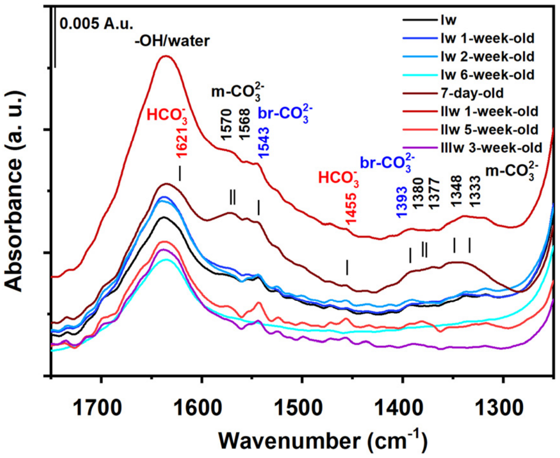

Figure S9. Structural changes of ***SZ3*** core@shell NPs after transfer into water by dialysis at pH 9 at different ages according to FTIR spectra. ***Iw*** (transfer on day 1 after zirconia deposition); ***IIw*** (transfer on day 7); ***IIIw*** (transfer on day 21). Hydrogenocarbonate/carbonate vibrational bands are slowly disappearing from the surface. The slowest desorption is observed for ***IIw***.

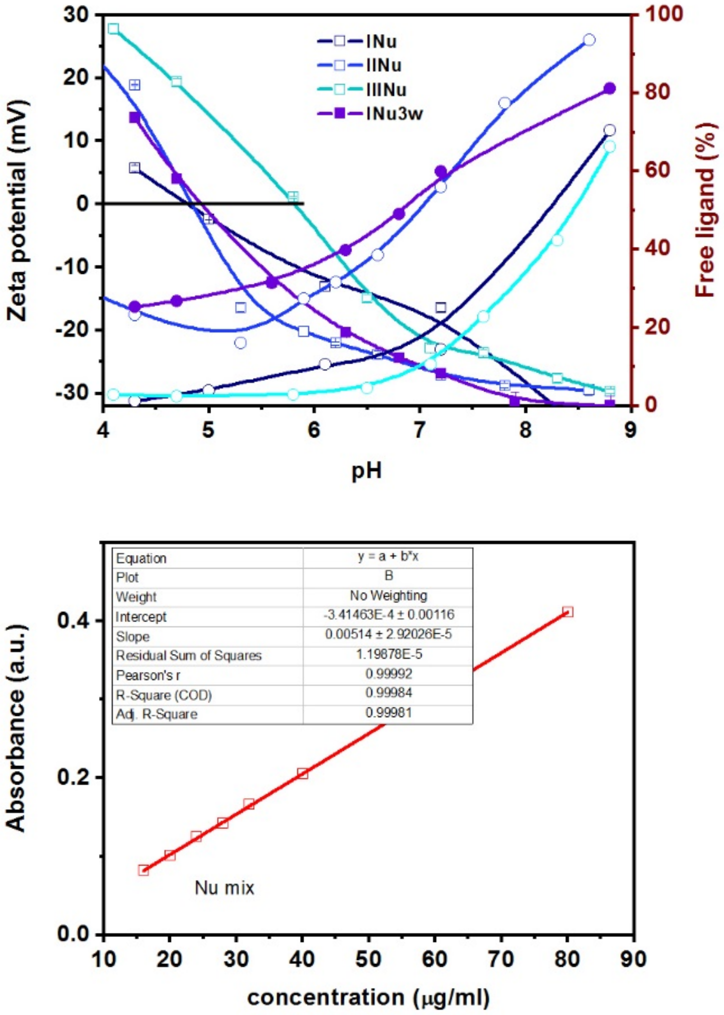

Figure S10. Combined zeta potential and free ligand quantity of the supernatant vs. pH curves obtained during desorption titration (upper) and calibration curve used for ***Nu*** mix quantity assessment (below).

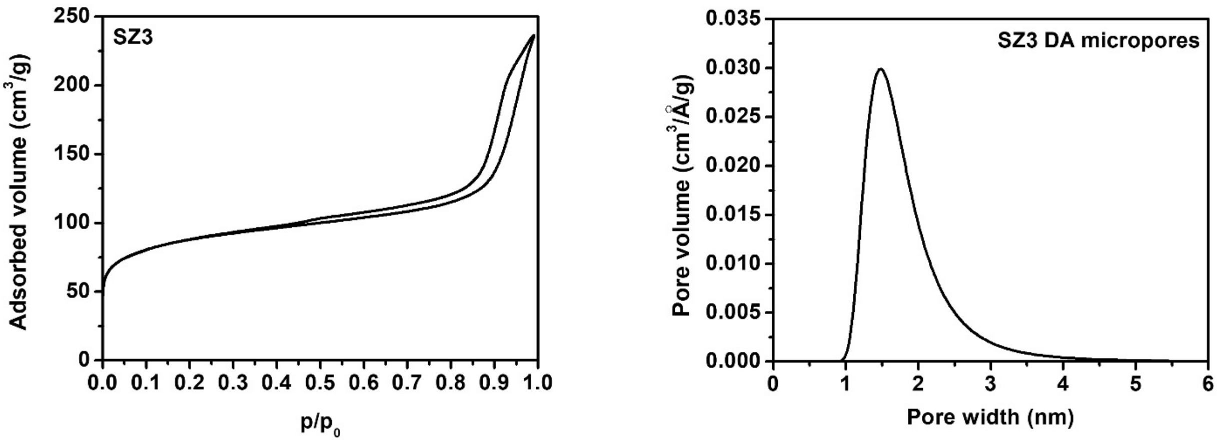

Figure S11. N_2 adsorption-desorption isotherm (left) and micropore size distribution (right) of ***SZ3*** (dried at the age of 4 days) powder.

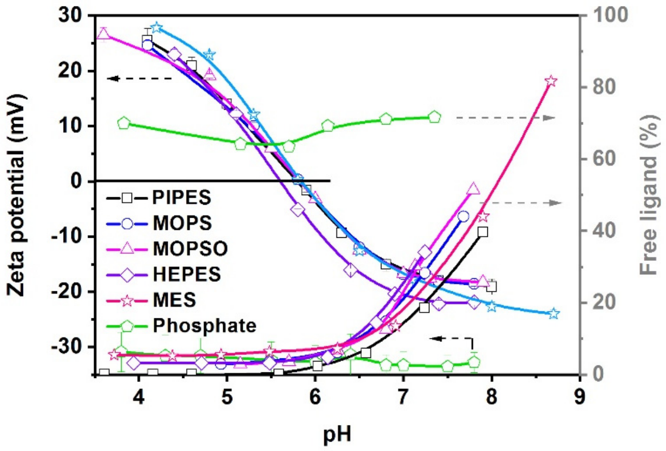

Figure S12. Variation of the zeta potential (left axis) and the percentage of free ligands (right axis) in the function of the pH for the dNMP mixture in the presence of 10 mM buffers.

Table S1. Goodness of fit and maximum absorbing dNMP mixture on ***IIw*** in 20 mM buffers at 22°C and at pH 7.2.

Buffer	R^2 value of the linear fit	a_m (mg/g)
**PIPES**	0.9991	110
**HEPES**	0.9993	187
**MOPS**	0.9908	119
**MOPSO**	0.9907	133
